# Epigenetic Alterations in Immune Cells of Systemic Lupus Erythematosus and Therapeutic Implications

**DOI:** 10.3390/cells11030506

**Published:** 2022-02-01

**Authors:** David E. Adams, Wen-Hai Shao

**Affiliations:** Division of Rheumatology, Allergy, and Immunology, Department of Internal Medicine, College of Medicine, University of Cincinnati, Cincinnati, OH 45267, USA; adamsdv@ucmail.uc.edu

**Keywords:** epigenetics, systemic lupus erythematosus, therapeutics, methylation, acetylation

## Abstract

Systemic lupus erythematosus (SLE) is an autoimmune disorder that is characterized by autoantibody production and dysregulated immune cell activation. Although the exact etiology of SLE remains unknown, genetic, hormonal, and complex environmental factors are known to be critical for pathologic immune activation. In addition to the inherited genetic predisposition, epigenetic processes that do not change the genomic code, such as DNA methylation, histone modification, and noncoding RNAs are increasingly appreciated to play important roles in lupus pathogenesis. We herein focus on the up-to-date findings of lupus-associated epigenetic alterations and their pathophysiology in lupus development. We also summarize the therapeutic potential of the new findings. It is likely that advances in the epigenetic study will help to predict individual disease outcomes, promise diagnostic accuracy, and design new target-directed immunotherapies.

## 1. Introduction

Systemic lupus erythematosus (SLE) is a female predominant autoimmune disorder characterized by autoantibody (autoAb) production and organ damages due to immune complex-mediated pathology [1]. SLE onset is believed to be triggered by environmental and hormonal factors in genetically susceptible individuals [2], but the exact cause remains unknown despite decades of intense basic and clinical research. Lupus flares are common, and induction and maintenance of remission are difficult to achieve. The mechanisms of lupus flare and remission are still being actively investigated by clinicians in the field [3]. Current treatments for SLE typically involve combinations of corticosteroids and immunosuppressant drugs, many of which are not FDA approved specifically for SLE and can be associated with significant side effects [4]. In over 50 years, however, only two new molecular targeted drugs recognizing B-cell activating factor (BAFF) (belimumab [5]) and IFN-I receptor (anifrolumab [6]) have been approved by FDA as add-on therapies for adult SLE patients.

The development of SLE involves deficiencies in both innate and adaptive immune systems. The innate immune system is a key player in perpetuating and amplifying the disease [7]. T cells play major roles in SLE pathogenesis, amplifying inflammation by their secretion of proinflammatory cytokines, helping B cells to generate autoAbs, and sustaining the disease through the accumulation of autoreactive memory T cells [8]. B cells, in turn, are the source of autoAbs, which trigger inflammatory cytokine production, abnormal transcription factor activity, and alter signaling pathways in affected organs [9]. In SLE development, it is believed that reoccurring environmental factors trigger proinflammatory responses in individuals with genomic and epigenomic susceptibility. Under such persistent inflammatory conditions, loss of the central and peripheral immunological tolerance leads to the clinical manifestation of SLE.

Although a recent powerful genome-wide association study (GWAS) has revealed over 100 lupus susceptible gene loci, thereby further improving our understanding of the genetic structure of SLE [2], gene abnormalities cannot fully account for the variety of lupus clinical manifestations. Epigenetic dysregulation further alters the susceptible molecular and cellular pathways attributed by the genomic variants and, thus, contributes to disease initiation and severity [10]. Epigenetic modifications of the most interest here refer to non-DNA sequence changes manifest at the DNA, RNA, or protein levels, including histone posttranslational modifications, DNA methylation, and alteration of gene expression by noncoding RNAs (ncRNAs) [11]. MicroRNAs (miRNAs) are noncoding small RNAs that act as epigenetic modulators to regulate the protein levels of target mRNAs without modifying the genetic sequences. Those inheritable changes are independent of the genomic DNA sequence. Epigenetic modifiers have been shown to control T/B cognate interactions during autoimmunity. In this review, we discuss recent findings on the epigenetic mechanisms of T- and B-cell activation and differentiation in the scope of SLE initiation and progression, and their involvement in lupus pathogenesis. Epigenetic modification targeted therapeutics are also highlighted.

## 2. T-Cell Epigenetic Alterations in SLE

T-cell dysregulation has been implicated in the loss of tolerance in SLE [12]. In general, the DNA of lupus CD4^+^ T cells is hypomethylated, which activates immune-related gene expression in a distinct CD4^+^ T-cell subtype and correlates with SLE disease activity. In principle, DNA hypomethylation can result from two different mechanisms: decreased methylation and increased demethylation activities. DNA methylation is catalyzed by DNA methyltransferases (DNMTs) [13]. Significantly lower DNMT1 and DNMT3A transcript levels were observed in SLE patients, compared with healthy controls [14]. Oxidative stress was shown to decrease DNMT1 levels and caused CD4^+^ T-cell gene activation in SLE patients [15,16]. DNA hypomethylation can also be achieved through active demethylation by a different set of enzymes. For example, 3-hydroxy butyrate dehydrogenase 2 (BDH2) is a short-chain dehydrogenase involved in maintaining intracellular iron homeostasis. In SLE CD4^+^ T cells, decreased BDH2 contributed to DNA hypomethylation via increasing intracellular iron [17]. The correlation between DNA hypomethylation and gene expression is largely based on broad gene association studies. Indeed, phenotypic differences seem to arise from diverse methylation patterns [18]. The impact of DNA methylations at distinct gene and CpG sites on SLE disease activity is not fully understood. Recently, a total of 22 CpG sites in the promoter and enhancer regions of the CD40 ligand gene (*CD40L*) were investigated for their functional association with the disease activity presented in 49 female SLE patients [19]. A site-specific hypomethylation of the *CD40L* promoter in CD4^+^ T cells was associated with SLE disease activity [19]. A genome-wide DNA methylation analysis also identified 55 differentially hypomethylated interferon-regulated genes in CD4^+^ T cells from twin SLE patients [20].

Histone acetylation and methylation contribute to the overexpression of immune-related genes that promote CD4^+^ T cell autoreactivity in SLE. In general, hypoacetylation seems to be negatively correlated and hypomethylation positively correlated with SLE disease activity [21]. However, conclusions were largely drawn from close association. It is not clear if these are secondary consequences or primary contributing factors in SLE. Nevertheless, among the core octamer histones, H3 modification (methylation, citrullination, or acetylation) seems to be predominant in SLE [22]. The trimethylation of histone H3 at lysine 27 (H3K27me3), in particular, leads to global gene silencing in animals, and increased H3K27me3 levels were reported in CD4^+^ T cells in lupus, compared with healthy controls [23]. In contrast, decreased H3K27me3 levels in the BCL6 promoter region led to significantly upregulated BCL6, which stimulates T_FH_ differentiation in SLE [24]. Molecularly, enhancer of zeste 2 polycomb repressive complex 2 subunit (Ezh2) is responsible for the trimethylation of H3K27. H3K27me3 and Ezh2 are critical for T-cell lineage development and activation [25]. Ezh2 expression is upregulated by TCR stimulation [26], and overexpression of Ezh2 resulted in increased CD4^+^ T-cell adhesion [23]. Inhibition of Ezh2 reduced STAT1 phosphorylation and IFN-I stimulated ISGs [27]. Ezh2-KO CD4^+^ T cells were arrested at early activation stages in OVA immunized mice [28]. Global hypoacetylation of histones H3 and H4 has also been detected in CD4^+^ T cells of active SLE patients, and the level of H3ac is negatively correlated with the disease activity [21]. Increased H3ac was found to couple with decreased H3K9me3 in the promoters of CD11a and CD70 in SLE CD4^+^ T cells [29].

ncRNAs are functional RNA molecules that are not translated into proteins. They are classified into long ncRNAs (lncRNAs, >200 nt) and short ncRNAs (sncRNAs, including miRNAs, <200 nt) [30]. Aberrant expression of ncRNAs and many miRNAs are observed in SLE and associated with disease severity [31]. Decreased expression of H/ACA box small nucleolar RNA 12 (SNORA12) was also found in SLE T cells in a small Taiwanese cohort (*n* = 23) study [32]. The levels of SNORA12 were inversely associated with higher SLE disease activity scores [32]. Numerous studies have revealed aberrant miRNA patterns in SLE patients and their likely involvement in SLE pathogenesis, including different cellular and molecular pathways. MiRNAs as disease biomarkers and therapeutic targets have been reviewed elsewhere [33,34,35].

Posttranscriptional modifications at the mRNA levels also serve as novel gene expression regulators. Studies have identified 78 hypomethylated 5-methylcytosine (m^5^C) transcripts and 131 hypermethylated transcripts in CD4^+^ T cells from Asian SLE patients. Hypermethylated genes were significantly involved in immune-related and inflammatory pathways, including interferon signaling [36], which is central to the pathogenesis of SLE [37]. A distinct regulation pattern of mRNA modifier, N4-acetylcytidine (ac^4^C) [38] and N6-methyladenosine (m^6^A) [39] has been observed in lupus and lupus nephritis, respectively, but the direct link with disease pathology has not been established. More studies are needed to relate this apparent correlation between RNA methylation and SLE to specific outcomes or signaling pathways in the pathology of SLE.

## 3. B-Cell Epigenetic Alterations in Lupus

B cells play crucial roles in lupus initiation and progression due to their contribution to cytokine secretion, antigen presentation, and autoAb production [40,41]. Interestingly, however, studies addressing lupus B-cell epigenetic modifications are fewer, compared with studies involving lupus T-cell epigenetics. Scharer et al. studied an African American (AA) SLE cohort with high disease activity and found the resting naïve B cells are epigenetically distinct in SLE [42]. In another study, the DNA methylation status (~460,000 CpG sites) of B cells in various development stages was examined in AA and European American (EA) SLE patients [43]. Epigenetic defects were identified in immature B cells from female AA patients with SLE, but defects developed later during B-cell development in EA female patients with SLE. AA-specific CpG sites are also enriched at the IFN-regulated genes (IRGs) [43]. Animal studies suggest that ten-eleven translocation (Tet) DNA methylase family members Tet2 and Tet3 mediated chromatin modification participated in the repression of CD86 on self-reactive B cells, a mechanism that may contribute to autoimmunity prevention. Indeed, Tet2 and Tet3 deficient B cells led to hyperactivation of B cells, autoAb production, and lupus-like disease in affected mice [44].

A more definitive comparison of environmental influences on SLE susceptibility would be to study identical twins. Such a study would minimize the genetic influences on SLE initiation, progression, and pathogenesis. For example, Ulff-Moller et al. studied the CpG methylation status in B cells from 15 SLE-affected twin pairs (6 homozygotic and 9 dizygotic). Predominantly hypermethylated CpG islands were observed in disease-associated B cells, and the most important upstream regulators included *TNF* and *EP300* [20]. Additionally, a global search of histone modification revealed that H3 and H4 are hypoacetylated in B cells from SLE patients [45]. Epigenetic regulation of abnormal X-linked gene expression also impacted the female disease susceptibility. Female lupus patients exhibited abnormal XIST long ncRNA localization, which resulted in 103 and 53 X-linked genes differentially expressed in naïve B cells and activated B cells, respectively [46].

## 4. SLE Therapeutics That Target Epigenetic Mechanisms

Epigenetic studies have enhanced our understanding of the pathogenic role of T and B cells in SLE. Epigenetic modifications are pharmaceutically reversible; therefore, targeted therapy is a feasible strategy in SLE. However, the clinical application of SLE epigenetics for precision medicine and therapeutic target discovery remains challenging. The first challenge is the global nature of the epigenetic modification. Histone and DNA CpG methylation–acetylation and ncRNA targeting are all multigene and noncell type specific. Therefore, therapeutic outcomes are hard to predict and may associate with adverse effects. Secondly, epigenetic alterations can represent a primary trigger for SLE pathogenesis or, alternatively, a secondary consequence of disease development. Such by-stander epigenetics change with the disease progression but do not reflect disease pathogenesis. Nevertheless, epigenetic aberrations have been proposed as diagnostic or disease prognostic markers [47]. On the other hand, if these challenges can be appropriately clarified, cell-specific epigenetic modulation may provide opportunities for therapeutic intervention. For example, Li et al. delivered 5-azacytidine (5-Aza) to T cells using nanolipogel-coated anti-CD4 or anti-CD8 Abs, resulting in expansion of Treg cells or decrease in DN T cells in MRL/*lpr* lupus-prone mice, respectively [48]. Ameliorated lupus manifestation was observed as a result [48].

A large number of miRNAs have been discovered to be associated with SLE pathogenesis through high throughput screening. Although therapeutics targeting miRNA are still at their early stage, preliminary results with lupus-prone mice are encouraging. Many miRNA targets, including miR-155, miR-146a, miR-21, miR-122, etc., have been shown to efficiently inhibit lupus disease development and renal inflammation in mouse models of lupus, and to suppress T-cell activation and T–B interaction [34,49]. miRNA-targeted therapy has been reviewed in detail by others [33,34].

The histone methyltransferase Ezh2 is currently under evaluation for the treatment of malignancy, and recent clinical trials have demonstrated a favorable outcome with Ezh2 inhibition [50]. Inhibition of Ezh2 by DZNep improved survival and significantly reduced renal inflammation in MRL/*lpr* spontaneous lupus mice before and after disease onset [51]. Inhibition of Ezh2 also attenuated the activation of the IFN-I signaling pathway [27]. However, DZNep is a global methyltransferase inhibitor, and adverse effects were associated with DZNep in animal models. Further studies in bm12-induced lupus and MRL/*lpr* spontaneous lupus with two structurally related Ezh2 selective small molecule inhibitors, GSK503 and GSK126, respectively, demonstrated significantly reduced autoAb production, GC formation, and improved lupus nephritis [27,28]. 

Targeting plasma cell differentiation and autoAb responses are proof-of-principle therapeutics in SLE. A preliminary study from the Casali group demonstrated histone deacetylase (HDAC) inhibitors (HDIs) valproic acid (VPA) and butyrate diminished plasma cell differentiation without altering B-cell viability and proliferation [52]. MRL/*lpr* lupus mice treated with HDI before or after disease onset showed significantly decreased anti-dsDNA titer and increased survival rate, compared with the control MRL/*lpr* mice [52]. Several other HDAC inhibitors have also been evaluated in mouse models of lupus. Their biological effects are summarized in Table 1.

Interestingly, the traditional SLE drug, mycophenolic acid (MPA, an immunosuppressant), was able to reverse the abnormal histone global hypoacetylation status in lupus CD4^+^ T cells by upregulating HAT expression and downregulating HDAC expression [53]. MPA also activates miR-142 and miR-146a [54], both of which have been reported to negatively regulate CD4^+^ T-cell activation in lupus [55,56,57]. Similarly, hydroxychloroquine (HCQ) or prednisone treated NZB/W spontaneous lupus mice showed reduced miR-21 and miR-let-7a expression in T and B cells, respectively [58]. Taken together, traditional SLE drugs may act as epigenetic modifiers.

## 5. Conclusions

Epigenetic processes in immune cells bridge the gap between genomics and environmental factors in the pathogenesis of SLE. Epigenetic alterations often couple with different cellular mechanisms to guide nuclear/cytoplasmic factors to mediate differential transcription/translation processes. A comprehensive understanding of the role of epigenetic modification in the already complex SLE pathogenesis will likely lead to safe and novel epigenetic therapeutics with better clinical outcomes.

## Figures and Tables

**Table 1:** Epigenetic therapeutics in mouse models of lupus and lupus nephritis.

Drug (Type)	Targets	Effects	Model	Ref.
ACY-738	HDAC6	↓ T- and B-cell development and response	MRL/*lpr* mice	[59]
			NZB/W mice	[60]
TSA	HDAC	↓ CD4^+^CD69^+^ T cells, ↑ CD4^+^CD25^+^ T_reg_ cells; ↓ IL-6, ↑TGF-β.	NZB/W mice	[61]
SAHA	HDAC	↓ cytokines, ↓ DN T cells	MRL/*lpr* mice	[62]
VPA	HDAC	↓ DN T cells	MRL/*lpr* mice	[63]
AZA nanolipogel	CD4 or CD8 T-cell-specific DNA demethylation	↑ T_reg_ cells, ↓ DN T cells	MRL/*lpr* mice	[48]
DZNep	Methyltransferase	↓ DN T cells, ↓ cytokine/chemokine	MRL/*lpr* mice	[51]
GSK503	Ezh2 methyltransferase	↓ T_FH_ cells	bm12 cGVHD	[28]
GSK126	Ezh2 methyltransferase	↓ IFN-I pathway	NZB/W mice	[27]

Notes: AZA, 5-azacytidine; DN, double negative; HDAC, histone deacetylase; SAHA, suberoylanilide hydroxamic acid; T_FH,_ follicular helper T cells; T_reg_, regulatory T cells; TSA, trichostatin A; VPA, valproic acid.

## Data Availability

Not applicable.
